# Perceptions and utilization of telemedicine for hair loss treatment: A survey-based analysis of Korean consumers

**DOI:** 10.1016/j.jpra.2025.10.003

**Published:** 2025-10-25

**Authors:** Jino Kim, Olena Sydorchuk, Kyuho Yi

**Affiliations:** a New Hair Plastic Surgery Clinic, Seoul, Korea; b SIDOR Inc., Kyiv, Ukraine; c Division in Anatomy and Developmental Biology, Department of Oral Biology, Human Identification Research Institute, BK21 FOUR Project, Yonsei University College of Dentistry, Seoul, Korea

**Keywords:** Telemedicine, Alopecia

## Introduction

Telemedicine has become integral to healthcare, a shift accelerated by the COVID-19 pandemic. In Korea, this extends to specialties like dermatology.^1^ However, its role in managing hair loss remains underexplored. Hair loss carries significant psychosocial weight and is a common concern in Korea. While traditionally managed through in-person visits, telemedicine offers a potential alternative to reduce access barriers. This study assesses the perceptions and utilization patterns of telemedicine for hair loss among Korean consumers.

## Material and method

We conducted a cross-sectional online survey from June 5 to July 1, 2024. We targeted members of a major Korean online hair loss community, collaborating with a digital health platform to recruit 697 adults aged 20+ with current or prior hair loss concerns. Established community members were prioritized to ensure response credibility.

The questionnaire covered demographics, treatment history, telemedicine use, barriers, motivations, and medication behaviors. Responses were anonymous, and electronic informed consent was obtained. Descriptive statistics were applied using SPSS version 28.0.

## Results

Among 697 respondents, 77.3 % were male and 52.7 % were in their thirties. Most lived in the Seoul metropolitan area and worked as office employees. A significant majority (91.0 %) had tried hair loss treatments. Despite this, only 31.2 % (*n* = 198) had used telemedicine for this purpose; 68.8 % (*n* = 436) had not.

The primary barrier was the belief that hair loss requires in-person evaluation (cited by >40 %). Other concerns included app usability and uncertainty about receiving prescriptions. However, 82.3 % of non-users were willing to try telemedicine in the future, motivated by expected lower costs, convenience, and improved accessibility.

Among pharmacological users, 77.4 % were currently on medication. Finasteride and dutasteride were most common. Most users indicated a preference for keeping monthly treatment costs below 30,000 KRW (∼22 USD).

## Discussion

Our findings reveal a significant gap between telemedicine availability and its adoption for hair loss in Korea. Skepticism about the adequacy of remote diagnosis for a condition often requiring visual and tactile assessment appears to be a key barrier, echoing broader concerns about tele-dermatology.^1^^,^^2^

Crucially, this study prompts a necessary discussion on the ethical and clinical implications of telemedicine as a sole modality for hair loss. While it offers undeniable benefits in accessibility, convenience, and potentially lower cost, it also carries risks. Remote consultations preclude a hands-on examination (e.g., pull test, scalp evaluation) which is critical for accurate differential diagnosis between androgenetic alopecia, alopecia areata, or scarring alopecias. The justification for its use may be strongest for follow-up care and prescription renewals in patients with established diagnoses rather than for initial evaluations.

The high willingness to consider future use suggests barriers are malleable. Educational initiatives clarifying the appropriate scope of tele-diagnosis, alongside improved app design and transparent pricing, could boost adoption, aligning with global trends where perceived benefit drives use.^3^^,^^4^

This study’s limitations include its online, self-selected sample, which may not be fully representative, and its cross-sectional design.

In conclusion, telemedicine for hair loss is underutilized in Korea, primarily due to diagnostic concerns. A hybrid model, combining initial in-person assessment with remote follow-up, may be most prudent. Addressing consumer skepticism through education and technology is key to unlocking its potential within Korea's dermatological landscape (Tables 1 and 2).

**Table 1:** Demographic characteristics of respondents.

Characteristic	Category	n (%)
Gender	Male	539 (77.3 %)
	Female	158 (22.7 %)
Age group	20s	124 (17.8 %)
	30s	367 (52.7 %)
	40s	161 (23.1 %)
	50 s and above	45 (6.5 %)
Region of residence	Seoul	237 (34.0 %)
	Gyeonggi Province	132 (18.9 %)
	Other regions	328 (47.1 %)
Occupation	Office worker	279 (40.0 %)
	Self-employed	72 (10.3 %)
	Professional	67 (9.6 %)
	IT worker	56 (8.0 %)
	Others	223 (32.0 %)

**Table 2:** Primary barriers to telemedicine use.

Barrier	n (%)
Belief that treatment must be face-to-face	227 (40.9 %)
Anticipated inconvenience with app usage	92 (16.6 %)
Concerns about medication pickup logistics	77 (13.9 %)
Anxiety about the reliability of telemedicine	63 (11.4 %)

## Declaration

This article does not contain any studies with human participants or animals performed by any of the authors.

## Ethical approval

This study was conducted in compliance with the principles set forth in the Declaration of Helsinki. All survey responses were anonymous and voluntary.

## Informed consent

Consent was received from the patient.

## Author contribution

Writing—Original Draft Preparation: Jino Kim; Kyuho Yi. Writing—Review & Editing: Jino Kim; Kyuho Yi, Olena Sydorchuk. Visualization: Jino Kim; Kyuho Yi. Supervision: Kyu-Ho Yi.

## Declaration of competing interest

I acknowledge that I have considered the conflict of interest statement included in the “Author Guidelines.” I hereby certify that, to the best of my knowledge, that no aspect of my current personal or professional situation might reasonably be expected to significantly affect my views on the subject I am presenting.

## References

[1] Seol J.E., Park S.H., Kim H. (2018). Analysis of live interactive teledermatologic consultations for prisoners in Korea for 3 years. J Telemed Telecare.

[2] Bourkas A.N., Barone N., Bourkas M.E.C. (2023). Diagnostic reliability in teledermatology: a systematic review and a meta-analysis. BMJ Open.

[3] Ahn J., Shin J., Lee J., Shin K., Park H. (2014). Consumer preferences for telemedicine devices and services in South Korea. Telemed J E-health : Official J Am Telemed Assoc.

[4] Santiago S., Lu J. (2023). Patient Satisfaction in teledermatology: an updated review. Curr Dermatol Rep.

